# Protective Effects of Baicalin on Decidua Cells of LPS-Induced Mice Abortion

**DOI:** 10.1155/2014/859812

**Published:** 2014-03-31

**Authors:** Xiaodan Wang, Yantao Zhao, Xiuhui Zhong

**Affiliations:** ^1^College of Traditional Chinese Veterinary Medicine, Agricultural University of Hebei, Baoding 071001, China; ^2^Institute of Traditional Chinese Veterinary Medicine, Agricultural University of Hebei, Baoding 071001, China

## Abstract

The study was carried out to investigate the protective effects of Baicalin on decidual cells of LPS-induced abortion mice. In the *in vitro* experiment, the decidual cells were cultured by uterus tissue mass cultivation sampled at day 6 of pregnancy, and gradient concentrations of LPS were used to determine the optimal LPS concentration of the injured decidual cells model. The injured decidual cells were treated with Baicalin (4 *μ*g/mL) to determine the protective role of Baicalin. In the *in vivo* experiment, lipopolysaccharide (LPS) was injected intravenously via the tail vein to induce abortion at day 6 of pregnancy, and the mice were given different concentrations of Baicalin by oral gavage consecutively at days 7 to 8 of pregnancy. On day 9 of gestation, the mice were sacrificed. The TNF and progesterone contents in the serum were assayed by ELISA. The results clearly revealed that Baicalin can prevent the injury to decidual cells from LPS dose dependently, TNF was decreased significantly (*P* < 0.01) compared to LPS group, and there was no effect on the progesterone. These findings suggest that Baicalin has protective effects on the injured decidual cells in the pregnant mice.

## 1. Introduction

Spontaneous abortions represent a common form of embryonic death caused by early pregnancy failure [1], and many factors are thought to be involved in this process, such as chromosomal and structural abnormalities and inflammation [2]. Most researches have focused on uterine decidual cells, which are known to play a major role in fetal development and survival. Decidua is the maternal tissue which depends on the levels of progesterone and estrogen in circulation for growth after blastocyst implants the endometrium. Decidua and trophoblast form the maternal portion of the placenta [3]. To protect the mother from the onslaught of invasive trophoblasts migrating toward the uterine spiral arteries, the endometrial stroma transforms itself into a dense cellular matrix known as the “deciduas” [4]. The formation of the decidua involves proliferation and differentiation. To accommodate the developing conceptus, a great number of decidua cells must die [5]. However, the overinjury of decidua cells will not be conductive to fetal nutrient supply and maintenance of immune microenvironment, which leads to the failure of pregnancy.

Bacterial LPS acting via toll-like receptors is required for early pregnancy failure in several murine abortion models [6]. Endotoxin (a component of the Gram-negative bacterial wall) is a potent inflammogen and is associated with the release of proinflammatory cytokines, such as IL-1, IL-6, and TNF [7]. Certain cytokines, such as IL-4, IL-6, and IL-10, seem to favor pregnancy success, whereas others, such as TNF and IFN-*γ*, are harmful to the embryo [8].

Baicalin is the main component of* Huang Qin* (*Radix scutellariae*), which has been commonly used in clinics as an antiabortive herb in China. Qin et al. [9] showed that Baicalin could make G0/G1 phase cells transition into the S phase and inhibit endometrial apoptosis induced by LPS. The reason for this may be that Baicalin can react antagonistically with the endotoxin LPS directly or decrease the proinflammatory cytokines. Some studies have demonstrated that Baicalin can adjust the Th1/Th2 cytokine balance and endocrine immune network, in the implantation and gestational periods [10].

Despite the existence of some reports on the antiabortive effects of Baicalin, only very limited research has been conducted into its mechanisms of protection on decidual cells against LPS. In the current experiment, we systematically investigated the role of Baicalin in protecting the decidual cells treated by LPS* in vitro* and* in vivo* and also its influence on the contents of TNF and progesterone.

## 2. Materials and Methods

### 2.1. Treatment of Animals

Eight-to-ten-week-old Kunming male and female mice were purchased from the Experimental Animal Center of Hebei Medical University, Shijiazhuang City, China. The mice were housed in a controlled environment (22~24°C), conditions of light (12 h light and 12 h darkness), and with free access to standard mouse food and water. Pregnancies were obtained by housing one female with one male; the females were examined each day in the early morning for the presence of a vaginal plug. The day that the vaginal plug was detected was designated as day 0 of pregnancy.

The mice were randomly divided into 5 groups at day 6 of pregnancy. The mice in group A were given PBS as the control group, and the mice in the other groups were injected intravenously with LPS at 0.1 mL/10 g via the lateral tail vein. Mice in groups C, D, and E received Baicalin at 0.25 mg/mL, 1.25 mg/mL, and 2.5 mg/mL (0.1 mL/10 g BW), respectively, on gestation days 7-8. Animals in groups A and B received an oral gavage of PBS on the same days of gestation as in group C. The animals were treated according to the Guidelines for Keeping Experimental Animals issued by the Chinese government.

On day 7 of gestation, the mice were sacrificed. The uterus was immediately weighed. The percentage of resorption was calculated by dividing the number of resorbed embryos by the number of implantations. The tissues were fixed in 4% paraformaldehyd. Paraffin-embedded tissue sections were cut at a thickness of 6 *μ*m, stained with hematoxylin and eosin, and examined under a light microscope.

Serum TNF and progesterone were measured by ELISA using commercial kits according to the manufacturer's instructions.

### 2.2. Chemicals and Reagents

Lipopolysaccharide (LPS) purchased from Sigma Co. (Saint Louis, MO, USA) was diluted to 1 ng/mL–1000 ng/mL with DMSO and then filtered and sterilized with a bacterial filter in the* in vitro* experiment and to 5 *μ*g/mL with 0.01 mol/L, pH 7.4, phosphate-buffered saline (PBS, filtered and sterilized with bacterial filter) in the* in vivo* experiment. Baicalin was purchased from the Chinese Institute for the Control of Pharmaceutical and Biological Products (Beijing, China). The agent was diluted with FD (F-12/DMEM = 1/1)-12 at concentrations of 4 mg/mL for the* in vitro* experiment. For the* in vivo *studies, Baicalin was dissolved with PBS to 0.25 mg/mL, 1.25 mg/mL, and 2.5 mg/mL. A progesterone kit and TNF ELISA kit were obtained from BD Biosciences (San Jose, CA, USA). FD-12 was obtained from Invitrogen (CA, USA). Fetal bovine serum (FBS) was obtained from Tianhang (Zhejiang, China).

### 2.3. Culture and Identification of Primary Decidual Cells

The mice were sacrificed at day 6 of pregnancy. Uterus tissue was obtained after removing the placentas and embryos and then rinsed with PBS to remove blood cells and mucus. The decidual tissue was placed into a small beaker with a small amount of culture medium. Afterwards, the decidual tissue was cut into small pieces of 0.5 mm³ and explanted in a 25 cm^2^ culture flask with 2 mL FD-12, containing 15% FBS. The cells were incubated at 37°C in a humidified atmosphere, with 5% CO_2_. The cells were passaged when attached to 80% of the culture flask. In order to identify the purity, we used the PRL (prolactin) immunohistochemistry kits to determine the percentage of positive decidua cells. The results showed that 90% of the cells were decidua cells; thus, we continued the drug treatment experiment.

### 2.4. Determination of the Optimum LPS Concentration

When the cultured cells occupied more than 80% of the culture flask, they were digested with 0.25% trypsin-EDTA, moved to 96-well plates with 1 × 10³ cells/cell, and treated with gradient concentrations of LPS (0 ng/mL, 1 ng/mL, 10 ng/mL, 100 ng/mL, 500 ng/mL, and 1000 ng/mL). The result was observed under a light microscope. The optimal LPS concentration used in the following experiment was determined with 50% decidual cell mortality.

To determine the effect of Baicalin, the attached cells were divided into a blank group, LPS group, and LPS + Baicalin (4 *μ*g/mL) group and incubated at 37°C in a humidified atmosphere, with 5% CO_2_; the cells' condition was observed after 48 h.

### 2.5. Statistical Analysis

The data were analyzed with SPSS 17.0 software and presented as average ± variance. Significant differences were compared among groups by one-way analysis of variance (ANOVA); *P* < 0.05 is determined to be significant and *P* < 0.01 is determined to be very significant.

## 3. Results

### 3.1. The Inhibition Effects of LPS on Decidua Cell Growth

The decidua cells were seen attached to the plate after a 24 h culture. The cells appeared with the morphology of fibroblasts or irregular stars; oval nuclei were located in the center, and nucleoli were clear with abundant cytoplasm granules. Shortly afterwards, new cells were generated surrounding the uterus blocks and then grew into monolayer cells after being cultured until 5 to 7 days. PRL identification result revealed that mice decidua cells were brown-stained, deeper, and more near to nucleoli.

**Figure 1:**
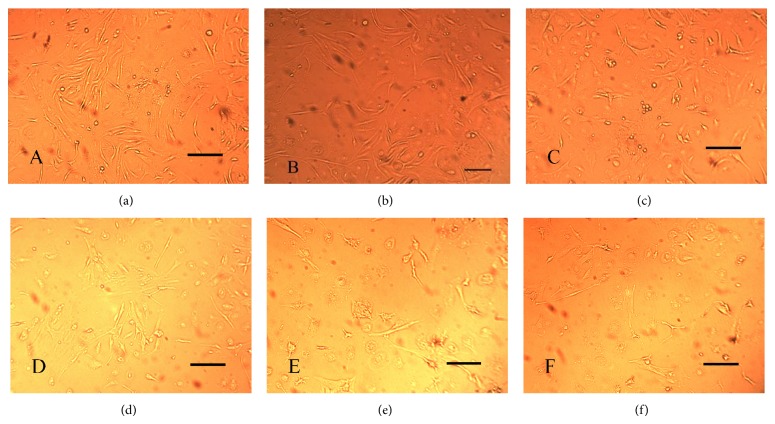
The inhibition effect of LPS on decidua cell growth. (a) 0 ng/mL LPS, the cells were normal, (b) 1 ng/mL LPS, very few cells were dead, (c) 10 ng/mL, few cells were dead, (d) 100 ng/mL LPS, 50% of the cells were dead, (e) 500 ng/mL LPS, most of the cells were dead, and (f) 1000 ng/mL, 98% of the cells were dead. The bars represent 100 *μ*m.

It was observed that the inhibition effect of LPS on decidua cell growth was seen starting from a concentration of 100 ng/mL. 500 ng/mL and 1000 ng/mL treatments led to serious damage to the cells, with a widening of the interspace between cells. Considering the vitality of the cells, a concentration of 100 ng/mL of LPS was selected (Figure 1).

### 3.2. The Protective Effects of Baicalin on Decidua Cells

**Figure 2:**
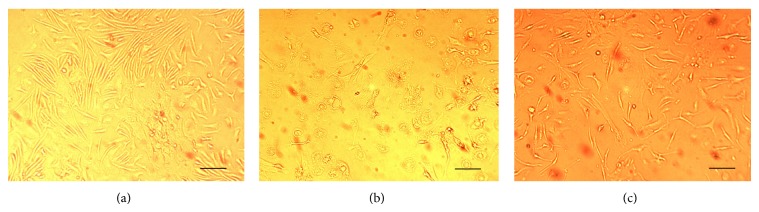
The role of Baicalin in survival of the decidua cells: (a) control group, (b) LPS group, and (c) Baicalin group. The bars represent 100 *μ*m.

Compared with the control group (A), the cell mortality rate of the LPS-treated model group (B) increased significantly. Moreover, after treatment with Baicalin, the survival rate of decidua cells increased compared to that of the model group. See Figure 2.

### 3.3. The Antiabortive Effects of Different Concentrations of Baicalin

**Figure 3:**
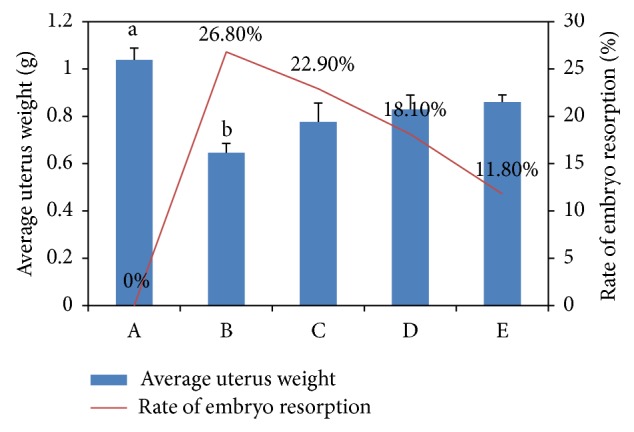
The weight and percentage of resorption: (A) control group, (B) LPS group, (C) 0.25 mg/mL Baicalin, (D) 1.25 mg/mL Baicalin, and (E) 2.5 mg/mL Baicalin.

The weight of the uteri of the LPS group was found to be significantly decreased compared with that of the control group (*P* < 0.01), and the embryo resorption rate of group B was increased to 26.8%. Mice in groups C to E, given different concentrations of Baicalin orally, showed increased uteri weights, and the embryo resorption rate decreased according to a dose-dependent relationship (Figure 3). The values of groups D and E are different significantly compared with group B (*P* < 0.05).

### 3.4. Histopathological Changes of the Uterus

**Figure 4:**
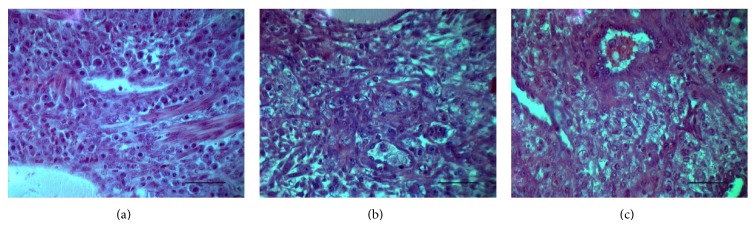
Histological analysis of decidual cells treated with Baicalin: (a) LPS group, (b) control group, and (c) Baicalin group. The bars represent 50 *μ*m.

In the control group (Figure 4(a)), the embryos were well oxygenated (pink) and showed a well-defined embryonic capsule and placenta. HE-straining showed that the nucleus was located in the center and dark-stained, and the vascular wall and glands were well developed; no hemorrhage or necrosis was observed. Compared with the control group, the model group (Figure 4(b)) embryos were usually smaller, showed signs of hemorrhage, and were without identifiable embryo or placenta. Decidua cell showed degeneration, hyperplasia, necrosis, and enlarged interstitial spaces. After supplementation with Baicalin (Figure 4(c)), the deciduas appeared thickened with the condition of alleviating hyperplasia and necrosis and with basic normal tissue structures.

### 3.5. Effects on Serum TNF and Progesterone

**Figure 5:**
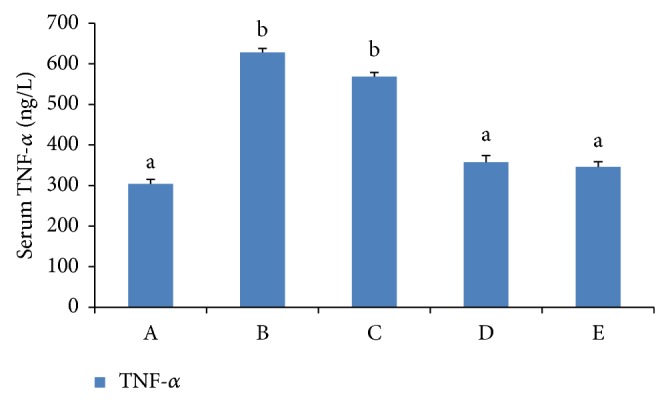
TNF in serum. Notes: (A) control group, (B) LPS group, (C) low dose of Baicalin (0.25 mg/mL), (D) middle dose of Baicalin (1.25 mg/mL), and (E) high dose of Baicalin (2.5 mg/mL). Different letters mean significant at 5% level, and same letters mean no significant difference.

**Figure 6:**
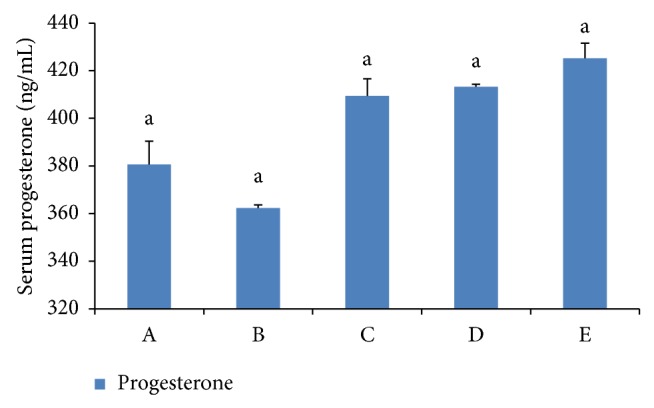
Progesterone in serum. Notes: (A) control group, (B) LPS group, (C) low dose of Baicalin (0.25 mg/mL), (D) middle dose of Baicalin (1.25 mg/mL), and (E) high dose of Baicalin (2.5 mg/mL). Different letters mean significant at 5% level, and same letters mean no significant difference.

The TNF level was increased compared with the PBS-treated mice in group A (*P* < 0.01). After Baicalin treatment (groups C, D, and E), the TNF levels were downgraded, and the degree of recovery depended on Baicalin concentrations. A better recovery degree was observed in 1.25 mg/mL and 2.5 mg/mL Baicalin groups (*P* < 0.01). In relation to progesterone, no significant difference was detected between groups (see Figures 5 and 6).

## 4. Discussion

Decidua is a fetomaternal interface containing multiple immunocytes, such as lymphocytes and macrophages, which play a central role in embryo embedding, placenta growth, and delivery [11]. LPS is the most potent antigenic component of the Gram-negative bacteria cell wall and is known to modulate the expression of various proinflammatory cytokines. The implantation sites are very sensitive to inflammation; thus, low doses of LPS do not endanger the survival of the mother but produce complete embryonic resorption (ER) after local activation of nitric oxide (NO) production [12]. LPS induced high levels of uterine NO production and has been reported to cause 100% embryonic resorption at 24 h, with complete fetus expulsions at 48 h [13]. Based on these findings, it can be assumed that LPS may induce damage to decidua cells directly or indirectly. To confirm this assumption, primary decidual cells obtained from day 6 of pregnant mice were treated with gradient concentrations of LPS for 24 h in the* in vitro* experiment; in the* in vivo* experiment, mice were treated with LPS at 0.1 mL/10 g by tail intravenous injection at day 6 of pregnancy. The results showed that LPS effects resulting in embryonic loss may be related to the necrosis in decidua cells.

Early embryo loss is closely related to the change of uterus immunological environment and hormone levels. Previous studies showed that exposure to* Escherichia coli *endotoxins contributed to the termination of pregnancy at any stage of gestation, but it did not disturb the normal endocrine function of pregnant mice [14]. LPS stimulates mononuclear macrophage, which produced an abundance of TNF and upregulated the TNF receptor [15]. LPS was not able to induce abortion without costimulation of TNF, indicating that simultaneous signals from multiple pathways are required [16]. TNF has been shown to perform a variety of biological functions, including the regulation of cell proliferation and the promotion of the trophoblast implanted to deciduas [17]. However, high levels of TNF have been shown to exert deleterious effects on pregnancy and impair trophoblast cell growth and functions* in vitro* [8]. High levels of TNF have been detected in spontaneous abortions woman than normal pregnancy woman in serum [18]. Progesterone (P) is the hormone of pregnancy and is unequivocally required in all mammals for maternal support of conceptus (embryo/fetus and associated membranes) survival and development [19]. The levels of circulating P below some undefined threshold or resistance of endometrium to otherwise adequate P will result in infertility or pregnancy loss [20]. Therefore, in the present study the levels of serum TNF and progesterone were detected. The results showed that LPS increased the level of serum TNF significantly (*P* < 0.01), leading to deciduas inflammation and immune responses, but the change of serum progesterone was not significant (*P* > 0.05). Our research was consistent with other reports.

Baicalin, a main active ingredient originally isolated from the root of* Huang Qin* (*Scutellaria baicalensis* Georgi), has a consistent record of safety in clinics and has been used as an anti-inflammatory drug in traditional Chinese medicine. Studies have shown that Baicalin could inhibit the proliferation of mononuclear cells, inhibit macrophage activation, and inhibit the production of Th1-related cytokines in different disease murine models [21]. Baicalin could improve the level of peripheral blood T lymphocyte subsets on drug-induced immunosuppressive mice, enhance the proportion of CD4+ and CD8+ cells of T lymphocytes, and regulate mice immunologic function [4]. Ma et al. [10] studies showed that Baicalin upregulated the level of progesterone in a dose-dependent manner, enhanced the secretion of IFN-*γ* during implantation, and downregulated IFN-*γ* production afterwards. These findings suggest that Baicalin can modulate the cytokine network in pregnancy and facilitate embryo implantation and survival. Previous studies in our laboratory have shown that Baicalin can adjust the Th1/Th2 cytokine balance and endocrine immune network in the implantation and gestational periods [22]. However, whether or not it can prevent LPS from increasing abortion rates has yet to be determined.

Compared with the LPS-model group, the cell mortality rate of the Baicalin-treated group was decreased significantly in the* in vitro* research. In the* in vivo* study, in which the pregnant animals at 7-8 days of pregnancy were treated with Baicalin, the rate of embryo resorption was decreased to 12%–23% in a dose-dependent manner; the serum TNF was downregulated significantly (*P* < 0.01) in the mice in the middle- and high-dose groups. It is suggested that the antiabortive effect of Baicalin on pregnant mice is related to the reduction of serum TNF, which is one of the main proinflammatory cytokines involved in abortion [23–25].

Our results also determined that the expression of progesterone was not changed significantly between the LPS group and the control group, with no apparent reference to the expression of progesterone after treatment with Baicalin. Whether all or part of the antiabortive effect of Baicalin is unrelated to serum progesterone remains to be investigated.

In summary, the results from the current study present a profile in which Baicalin has a dose-dependent protective effect on damaged deciduas cells by LPS via regulation of the level of TNF in serum. Nevertheless, the specific mechanism of the antiabortive effect of Baicalin needs further study.
